# Correlation between Buruli Ulcer and Vector-borne Notifiable Diseases, Victoria, Australia

**DOI:** 10.3201/eid1504.081162

**Published:** 2009-04

**Authors:** Paul D.R. Johnson, Caroline J. Lavender

**Affiliations:** Austin Health, Melbourne, Victoria, Australia (P.D.R. Johnson); World Health Organization Collaborating Centre for *Mycobacterium ulcerans* (Western Pacific Region); Victorian Infectious Diseases Reference Laboratory, North Melbourne, Victoria, Australia (P.D.R. Johnson, C.J. Lavender)

**Keywords:** Australia, Buruli ulcer, mosquitoes, notifiable diseases, skin disease, Ross River virus, Barmah Forest virus, letter

**To the Editor:** Buruli ulcer (BU) is a destructive skin disease caused by the toxin-producing environmental pathogen *Mycobacterium ulcerans*. Since the 1980s, BU has emerged as a major public health problem in rural West and Central Africa (*1*), where some researchers have suggested a role for aquatic insects as either reservoirs or vectors of *M. ulcerans* (*2*,*3*). However, this hypothesis remains unproven (*4*).

In contrast to the emerging BU–endemic areas in tropical rural West Africa, the climate of the Australian state of Victoria is temperate, yet locally acquired BU also has increased there in recent years (*5*). In addition, notifications have varied markedly from year to year for reasons not yet explained.

During the investigation of a new outbreak of BU in Victoria, we demonstrated that *M. ulcerans* is detectable by PCR in mosquitoes and that being bitten by mosquitoes increases the odds of being diagnosed with BU (*6*,*7*). However, *M. ulcerans*–positive mosquitoes might reflect only the presence of *M. ulcerans* in the local environment and play no role in transmission. To further investigate links between BU and mosquitoes, we compared patterns of notifications of BU with other notifiable diseases in Victoria. In particular, we were interested in any association between BU and the locally transmitted vector-borne alphaviruses Ross River virus (RRV) and Barmah Forest virus (BFV). Areas of BU and RRV/BFV endemicity overlap geographically, but areas with RRV and BFV are more extensive and include inland river systems where BU has not so far been reported.

Notification data for RRV, BFV, and other notifiable infections in Victoria are publicly available (*8*). Although BU was not made notifiable until January 2004 (before which notification was voluntary), since early 2000, most diagnoses were confirmed by culture or PCR at the Victorian Infectious Diseases Reference Laboratory, from which we obtained data for this report.

Our analysis showed that in the last 7 years (2002–2008), BU notifications correlated with combined RRV/BFV notifications (r^2^ = 0.52, p = 0.06) (Figure). During the same period, no correlation was observed with tuberculosis, the other important mycobacterial disease in Victoria (r^2^ = 0.12, p = 0.43); legionellosis, caused by a nonvectored water-associated pathogen (r^2^ = 0.04, p = 0.66); or any other notifiable infectious disease (data not shown).

**Figure:**
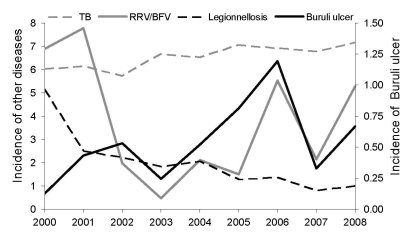
Numbers of cases per 100,000 inhabitants for selected notifiable diseases, Victoria, Australia, 2000–2008. Buruli ulcer is shown on the right y axis, other diseases on the left y axis. RRV, Ross river virus; BFV, Barmah Forest virus; TB, tuberculosis.

Although the environmental reservoir and mode of transmission of *M. ulcerans* remain unknown, mosquitoes are well known for transmitting RRV and BFV to humans, and year-to-year variation in incidence of these vector-borne viral infections is linked to changes in mosquito numbers (*9*,*10*). We are not implying that *M. ulcerans*, RRV, and BFV are transmitted simultaneously from the same reservoir species to the same humans or by the same mosquitoes. Also, environmental conditions that promote outbreaks of RRV/BFV infection might promote BU outbreaks without any other connection. However, we believe the correlation we have identified between BU and other mosquito-borne diseases is striking and further strengthens the link between mosquitoes and the transmission of *M. ulcerans* in Victoria.
